# *SnakeMAGs*: a simple, efficient, flexible and scalable workflow to reconstruct prokaryotic genomes from metagenomes

**DOI:** 10.12688/f1000research.128091.1

**Published:** 2022-12-15

**Authors:** Nachida Tadrent, Franck Dedeine, Vincent Hervé

**Affiliations:** 1 Institut de Recherche sur la Biologie de l'Insecte, UMR 7261, CNRS-Université de Tours, Tours, 37200, France; 2 Université Paris-Saclay, INRAE, AgroParisTech, UMR SayFood, Palaiseau, 91120, France

**Keywords:** Snakemake, metagenomics, microbiology, genomics, bioinformatics, microbial ecology

## Abstract

**Background: **Over the last decade, we have observed in microbial ecology a transition from gene-centric to genome-centric analyses. Indeed, the advent of metagenomics combined with binning methods, single-cell genome sequencing as well as high-throughput cultivation methods have contributed to the continuing and exponential increase of available prokaryotic genomes, which in turn has favored the exploration of microbial metabolisms. In the case of metagenomics, data processing, from raw reads to genome reconstruction, involves various steps and software which can represent a major technical obstacle.

**Methods: **To overcome this challenge, we developed *SnakeMAGs*, a simple workflow that can process Illumina data, from raw reads to metagenome-assembled genomes (MAGs) classification and relative abundance estimate. It integrates state-of-the-art bioinformatic tools to sequentially perform: quality control of the reads (illumina-utils, Trimmomatic), host sequence removal (optional step, using Bowtie2), assembly (MEGAHIT), binning (MetaBAT2), quality filtering of the bins (CheckM), classification of the MAGs (GTDB-Tk) and estimate of their relative abundance (CoverM). Developed with the popular Snakemake workflow management system, it can be deployed on various architectures, from single to multicore and from workstation to computer clusters and grids. It is also flexible since users can easily change parameters and/or add new rules.

**Results: **Using termite gut metagenomic datasets, we showed that *SnakeMAGs* is slower but allowed the recovery of more MAGs encompassing more diverse phyla compared to another similar workflow named ATLAS.

**Conclusions: **Overall, it should make the reconstruction of MAGs more accessible to microbiologists. *SnakeMAGs* as well as test files and an extended tutorial are available at https://github.com/Nachida08/SnakeMAGs.

## Introduction

Over the last years, microbial ecology has progressively made the transition from gene-centric to genome-centric analyses, ^1^ allowing the clear assignment of (sometimes novel) microbial taxa to specific functions and metabolisms. ^2^^–^^5^ Indeed, technical and technological progresses such as binning methods applied to metagenomics, ^6^ single-cell genome sequencing ^7^ as well as high-throughput cultivation methods ^8^ have contributed to the continuing and exponential increase of available prokaryotic genomes. ^9^ This is particularly true for metagenomics that offers the possibility to reconstruct metagenome-assembled genomes (MAGs) on a large scale and from various environments, and thus has generated a huge amount of new prokaryotic genomes. ^10^^,^^11^

Although the use of MAGs in microbial ecology is becoming a common practice nowadays, processing raw metagenomic reads up to genome reconstruction involves various steps and software which can represent a major technical obstacle, especially for non-specialists. To face this problem, several workflows such as MetaWRAP, ^12^ its Snakemake version called SnakeWRAP, ^13^ ATLAS ^14^ and more recently MAGNETO, ^15^ have been developed to automatically reconstruct genomes from metagenomes. However, these workflows contain various modules and perform more tasks than only generating MAGs. For instance, they will taxonomically assign the metagenomic reads, create gene catalog or perform functional annotations. They rely on numerous dependencies, require significant computational resources and regenerate a lot of outputs which are not essential to most research projects. To simplify this procedure and make it more accessible while remaining efficient, reproducible and biologically relevant, we developed with the popular Snakemake workflow management system, ^16^ a configurable and easy-to-use workflow called *SnakeMAGs* to reconstruct MAGs in just a few steps. It integrates state-of-the-art bioinformatic tools to sequentially perform from Illumina raw reads: quality filtering of the reads, adapter trimming, an optional step of host sequence removal, assembly of the reads, binning of the contigs, quality assessment of the bins, taxonomic classification of the MAGs and estimation of the relative abundance of these MAGs.

## Methods

### Creation

Our tool was built by integrating a set of software needed to process metagenomic datasets, utilizing Snakemake. There are no additional equations/maths needed to recreate this tool.

### Implementation

The workflow has been developed with the workflow management system Snakemake v7.0.0 ^16^ based on the Python language. Snakemake enables reproducible and scalable data analyses as well as an independent management of the required software within a workflow. *SnakeMAGs* is composed of two main files:

The Snakefile, named “SnakeMAGs.smk”, contains the workflow script. It is divided into successive rules which correspond to individual steps. Our workflow includes a total of 15 distinct rules. Each rule requires input files and relies on a single software installed independently when starting the workflow in a dedicated conda v4.12.0 environment. At the end of each rule, output files will be generated in a dedicated folder, as well as a log file (stored in the logs folder) summarizing the events of the software run and a benchmark file (stored in the benchmarks folder) containing the central processing unit (CPU) run time, the wall clock time and the maximum memory usage required to complete the rule. Thanks to Snakemake wildcards, our rules are generalized, so one can process multiple datasets in parallel without having to adjust the source code manually.

The configuration file, ^40^ named “config.yaml”, is used to define some variable names ( *e.g.* names of the input files), paths ( *e.g.* working directory, location of the reference databases), software parameters and computational resource allocations (threads, memory) for each of the main steps.

To run the workflow, the user only requires Snakemake. It can be easily installed, for instance *via* Conda, as explained in the GitHub repository:



conda create -n snakemake_7.0.0 snakemake=7.0.0


After that, the user will only have to edit the config file (an example is provided on the GitHub repository) and then run *SnakeMAGs*:



#Example of command on a Slurm cluster
snakemake --snakefile SnakeMAGs.smk --cluster \
'sbatch -p <cluster_partition> --mem -c \ 
-o "cluster_logs/{wildcards}.{rule}.{jobid}.out" \ 
-e "cluster_logs/{wildcards}.{rule}.{jobid}.err" ' \ 
--jobs --use-conda --conda-frontend \
conda --conda-prefix/path/to/SnakeMAGs_conda_env/ \
--jobname "{rule}.{wildcards}.{jobid}" --configfile/path/to/config.yaml


During the first use of the workflow, a dedicated Conda environment will be installed for each of the bioinformatic tool to avoid conflict. Then the input files will be processed sequentially. Output files will be stored in eight dedicated folder: logs, benchmarks, QC_fq (containing FASTQ files), Assembly, Binning, Bins_quality (all three containing FASTA files), Classification (containing FASTA files and text files with the taxonomic information), and MAGs_abundances (text files).

The workflow has been successfully used on a workstation with Ubuntu 22.04 as well as on high-performance computer clusters with Slurm v18.08.7 and SGE v8.1.9.

### Operation

The minimal system requirements to run the workflow will depend on the size of the metagenomic dataset. Small datasets ( *e.g.* the test files provided on the GitHub repository) have been successfully analyzed on a workstation with an Intel Xeon Silver 4210, 2.20GHz (10 cores/20 threads) processor and 96GB of RAM. Larger datasets should be processed on cluster computing or within a high-performance infrastructure. For instance, performance evaluation of publicly available metagenomes (see below) was performed on a computer cluster under CentOS Linux release 7.4.1708 distribution with Slurm 18.08.7, on a node possessing an Intel Xeon CPU E7-8890 v4, 2.20GHz (96 cores/192 threads) and 512 GB RAM.

**Figure 1.**
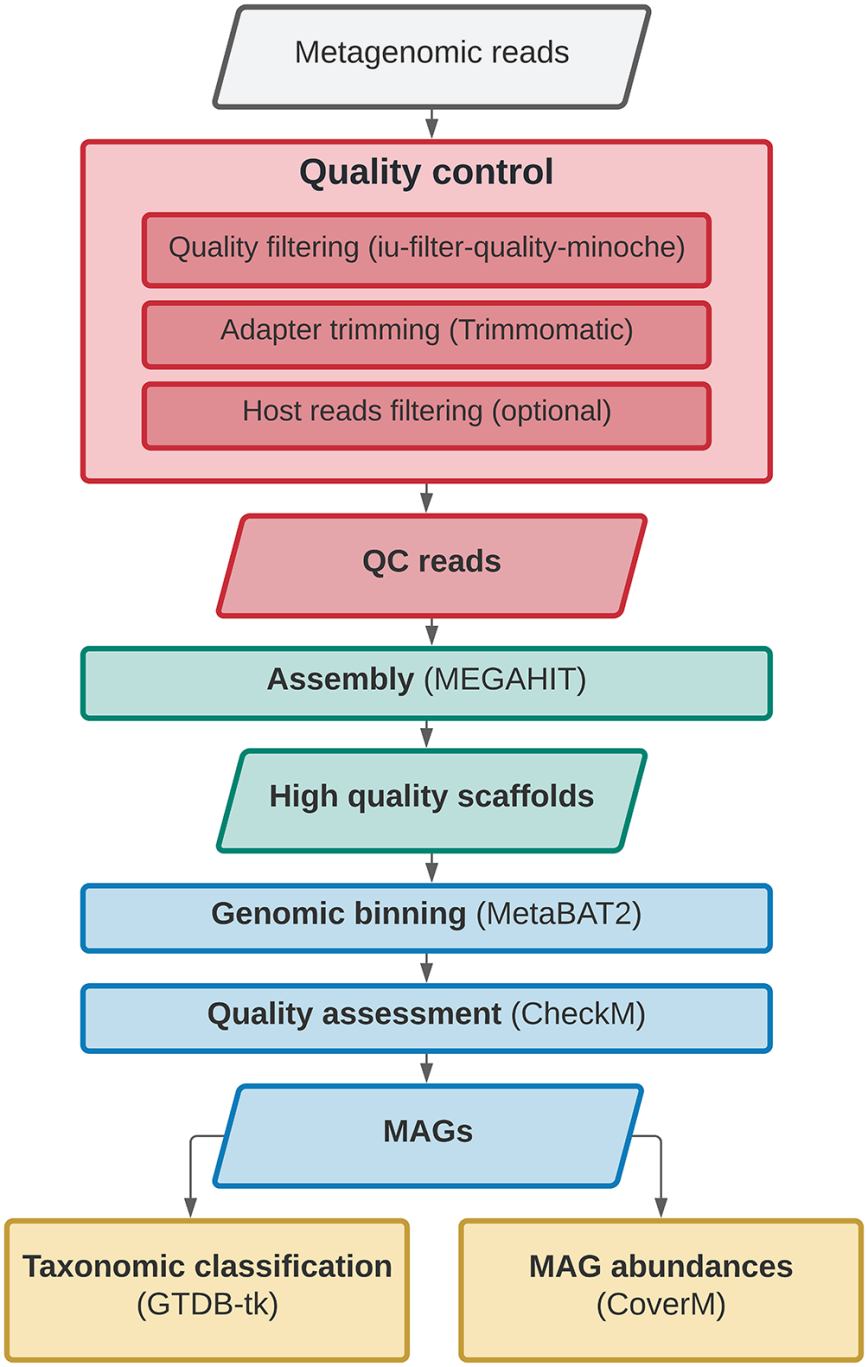
Directed acyclic graph describing the main steps performed by *SnakeMAGs.* The names of the software used for each step are showed in parentheses.

*SnakeMAGs* integrates a series of bioinformatic tools to sequentially perform from Illumina raw reads: quality filtering of the reads with illumina-utils v2.12, ^17^ adapter trimming with Trimmomatic v0.39 ^18^ (RRID:SCR_011848), an optional step of host sequence removal ( *e.g.* animal or plant sequences) with Bowtie2 v2.4.5 ^19^ (RRID:SCR_016368), assembly of the reads with MEGAHIT v1.2.9 ^20^ (RRID:SCR_018551), binning of the contigs with MetaBAT2 v2.15 ^21^ (RRID:SCR_019134), quality assessment of the bins with CheckM v1.1.3 ^22^ (RRID:SCR_016646), classification of the MAGs with GTDB-Tk v2.1.0 ^23^ (RRID:SCR_019136) and estimation of the relative abundance of these MAGs with CoverM v0.6.1. An overview of the workflow is presented in Figure 1.

## Use cases

To demonstrate the benefits and potential of our workflow, we compared it to another Snakemake workflow named ATLAS v2.9.1. ^14^ To produce a fair comparison, ATLAS was run with the MEGAHIT assembler, without co-binning and dereplicating only 100% similar MAGs. To test these two workflows, we downloaded and analyzed ten publicly available termite gut metagenomes (accession numbers: SRR10402454; SRR14739927; SRR8296321; SRR8296327; SRR8296329; SRR8296337; SRR8296343; DRR097505; SRR7466794; SRR7466795) from five studies ^24^^–^^28^ and belonging to ten different termite species.

*SnakeMAGs* requires only a limited number of inputs files: the raw metagenomic reads in FASTQ format from the 10 above-mentioned metagenomes, a FASTA file containing the adapter sequences, ^40^ a YAML configuration file specifying the variable names, paths and computational resource allocations (available on the GitHub repository and on Zenodo), and here since we worked with host-associated metagenomes a FASTA file containing the termite genome sequences. ^39^ Regarding the outputs, *SnakeMAGs* produced quality-controlled FASTQ files without adapters nor termite sequences, in the QC_fq folder. Then the reads assembled into contigs and scaffolds (FASTA files) were saved in the Assembly folder. Products of the binning procedure were stored in the Binning folder. Bins with >50% completeness and <10% contamination (according to CheckM) were considered as MAGs and stored in the Bins_quality folder. Subsequently, the results of the MAGs classification and relative abundance estimation were sent to the Classification and MAGs_abundances folders, respectively. ATLAS requires similar input files and produces, among others, similar outputs files.

**Figure 2.**
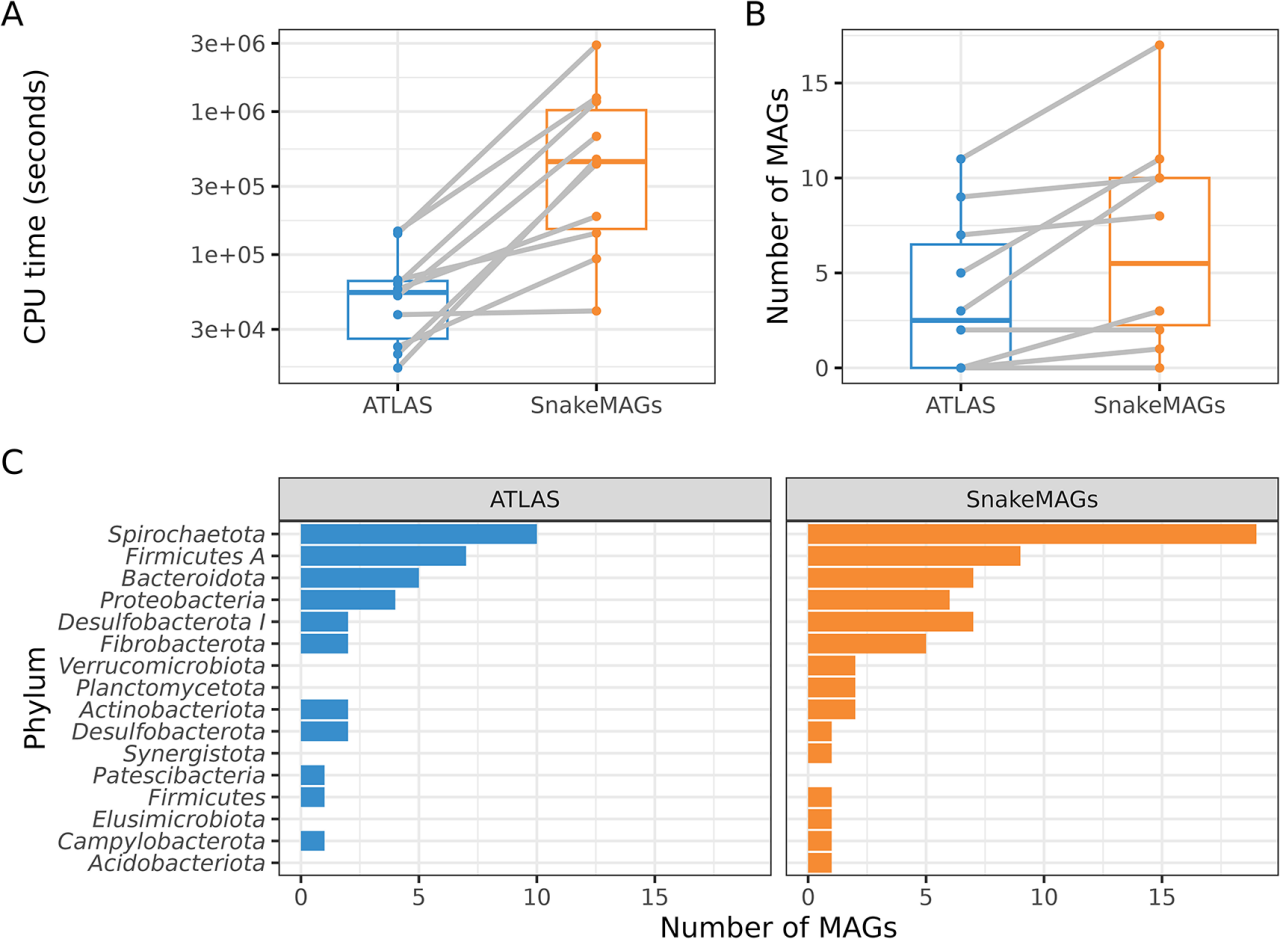
Comparison of the performance of *SnakeMAGs* v1.0.0 with another workflow, namely ATLAS v2.9.1 ^14^ using 10 termite gut metagenomes. A. CPU time (in seconds) required to process each metagenome. B. Number of MAGs reconstructed from each metagenome. On both boxplots, gray lines link the result obtained with ATLAS and the one obtained with *SnakeMAGs* for each of the 10 analyzed termite metagenomes. C. Number of bacterial MAGs at the phylum level recovered from each workflow.

ATLAS appeared to be faster than *SnakeMAGs* to reconstruct MAGs from metagenomes ( Figure 2A). However, *SnakeMAGs* always recovered more MAGs (>50% completeness and <10% contamination according to CheckM) per metagenome or at least as much as ATLAS ( Figure 2B). From the ten metagenomes, *SnakeMAGs* produced a total of 65 MAGs while ATLAS generated only 37 MAGs. Additionally, *SnakeMAGs* was able to recover MAGs encompassing a higher diversity of bacterial phyla ( *n* = 15 phyla) compared to ATLAS ( *n* = 11 phyla). Only one phylum, namely *Patescibacteria*, represented by a single MAG was recovered by ATLAS and not by *SnakeMAGs.* On the contrary, ATLAS failed to reconstruct MAGs belonging to *Verrucomicrobiota*, *Planctomycetota*, *Synergistota*, *Elusimicrobiota* and *Acidobacteriota* when *SnakeMAGs* succeeded ( Figure 2C).

## Discussion

Using metagenomic datasets from the gut of various termite species, our analyses revealed that while being slower, *SnakeMAGs* allowed the recovery of more MAGs encompassing more diverse phyla compared to ATLAS, another similar Snakemake workflow. More importantly our results showed that *SnakeMAGs* was able to recover MAGs encompassing the major bacterial phyla found in termite guts, ^29^^,^^30^ and that some of these phyla were not recovered by ATLAS. Indeed, taxa belonging to *Verrucomicrobiota,* ^31^ *Planctomycetota,* ^30^^,^^32^ *Synergistota,* ^33^ *Elusimicrobiota* ^34^ and *Acidobacteriota* ^35^^,^^36^ have been repeatedly found in the gut of various termite species. As such, they would represent relevant targets for genome-centric analyses of the termite gut microbiota. Therefore, we showed that *SnakeMAGs* has the potential to retrieve quantitatively more genomic information from metagenomes but also to extract genomic features of biological interest.

Thanks to the inherent flexibility of Snakemake, *SnakeMAGs* offers the possibility to the users to easily tune the parameters of the workflow ( *e.g.* resource allocations for each rule, options of a specific tools) to adapt their analysis to the datasets and to the computational infrastructure. Additionally, advanced users will have the opportunity to edit or add new rules to the workflow. Regarding the future of *SnakeMAGs*, several avenues will be considered for the next versions of the workflow. Firstly, the workflow could give more freedom to the users by offering the choice of different tools to perform the same task ( *e.g.* different trimming, assembly or binning software). Secondly, with the current emergence of metagenomic datasets generated with long-read DNA sequencing, ^37^ it might be relevant to adjust our workflow for long-read sequencing technology by including specific bioinformatic tools for this technology. ^38^ Meanwhile, since the majority of the metagenomic datasets have been and are still currently generated with Illumina short-read technology, *SnakeMAGs* can be widely used to explore the genomic content of various ecosystems *via* metagenomics.

## Software availability

Source code available from: https://github.com/Nachida08/SnakeMAGs

Archived source code at time of publication: https://doi.org/10.5281/zenodo.7334838. ^39^

License: CeCILL v2.1

## Data Availability

Termite genome references used for removing host sequences and their Bowtie2 index are available at: https://zenodo.org/record/6908287#.Y1JLANJBzUR The termite gut metagenomes analyzed in the present study are available on NCBI with the following accession numbers: SRR10402454; SRR14739927; SRR8296321; SRR8296327; SRR8296329; SRR8296337; SRR8296343; DRR097505; SRR7466794; SRR7466795. Zenodo. Reconstruction of prokaryotic genomes from ten termite gut metagenomes using two distinct workflows: SnakeMAGs and ATLAS: https://doi.org/10.5281/zenodo.7334397. ^40^
- SnakeMAGs_config.yaml (The configuration file used to analyze the 10 termite gut metagenomes with *SnakeMAGs*) - ATLAS_config.yaml (The configuration file used to analyze the 10 termite gut metagenomes with ATLAS) - MAGs_SnakeMAGs.zip (A zipped folder containing the genomes of the 65 MAGs reconstructed with *SnakeMAGs*) - MAGs_ATLAS.zip (A zipped folder containing the genomes of the 37 MAGs reconstructed with ATLAS) -
taxonomic_assignment_MAGs.csv (A text file containing the taxonomic assignment of all the MAGs reconstructed by both workflows) Data are available under the terms of the Creative Commons Attribution 4.0 International license (CC-BY 4.0).
