# Supplementary material for: SnakeMAGs: a simple, efficient, flexible and scalable workflow to reconstruct prokaryotic genomes from metagenomes
Source: F1000Res. 2023 Feb 27;11:1522. Originally published 2022 Dec 15. [Version 2] doi: 10.12688/f1000research.128091.2 (PMC9978240; doi:10.12688/f1000research.128091.2)
Supplement: Peer review history

# Peer review: Reviewer response for version 2

**DOI:** 10.5256/f1000research.144793.r164770

**Published:** 2023-03-01

**Authors:** Célio Dias Santos Júnior

**Affiliations:** 1 Institute of Science and Technology for Brain-Inspired Intelligence - ISTBI, Fudan University, Shanghai, China

The new improvements implemented in the paper " *SnakeMAGs*: a simple, efficient, flexible and scalable workflow to reconstruct prokaryotic genomes from metagenomes" not only rise its scientific value but also supply strong evidence of its applicability and importance. I feel convinced by the answers and additional analysis provided by Tradent *et al.* and praise the authors for their disposition in implementing some of my suggestions, especially about the release of a new version containing GUNC as a filter.

Are the conclusions about the tool and its performance adequately supported by the findings presented in the article?

Partly

Is the rationale for developing the new software tool clearly explained?

Partly

Is the description of the software tool technically sound?

Yes

Are sufficient details of the code, methods and analysis (if applicable) provided to allow replication of the software development and its use by others?

Yes

Is sufficient information provided to allow interpretation of the expected output datasets and any results generated using the tool?

Yes

Reviewer Expertise:

Bioinformatics, microbiology, biochemistry, biotechnology

I confirm that I have read this submission and believe that I have an appropriate level of expertise to confirm that it is of an acceptable scientific standard.

# Peer review: Reviewer response for version 1

**DOI:** 10.5256/f1000research.140650.r159904

**Published:** 2023-02-15

**Authors:** Aram Mikaelyan

**Affiliations:** 1 Department of Entomology and Plant Pathology, North Carolina State University, Raleigh, NC, USA

The authors describe SnakeMAGs as a dedicated pipeline for the compositional binning of contigs generated from Illumina sequencing reads obtained from microbial communities. It integrates the various steps in the process of comparative analyses in metagenomes, starting with the assembly of reads generated from community DNA, to the compositional binning of the assembled contigs into high-quality metagenome assembled contigs (MAGs). I believe this pipeline represents a valuable addition to the community. I found the manuscript was easy to read and have the following comments for the authors:

1. The main advantage of Snake is that it is highly task-specific to the generation of high-quality MAGs from complex communities. It therefore can save considerable time and resources by avoiding steps in processing data (e.g. extensive functional or taxonomic annotations) that are irrelevant to the researcher’s objective.
  I would like the authors to expand on how their pipeline controls for chimeric sequences, since these may lead to an artefactual inflation of MAGs.
2. As a termite researcher, I am aware of the many quirks that termite gut microbiomes present, and the limitations of many data processing pipelines and databases when it comes to addressing those quirks. I was glad that the authors used this challenging community for their benchmarks, and impressed at the contrast between the results from SnakeMAGs and ATLAS. I will emphasize further that the apparent inability of ATLAS to report phyla such as Planctomycetota and Elusimicrobia, or reconstruct fewer MAGs from Fibrobacterota or Desulfobacterota, is not trivial. These bacterial members play major roles in the microbial ecology of the termite gut, and the SnakeMAGs’ ability to detect them highlights its superiority.
  I would like the authors to list/explain some factors could potentially account for this difference.
3. I found the installation and usage on a Linux Mint server (with comparable processing power to the one used by the authors) to be straightforward. The dependencies are minimalistic and easy to install.

I congratulate the authors on sharing this tool with the community and hope they find my comments useful.

 Aram Mikaelyan

Are the conclusions about the tool and its performance adequately supported by the findings presented in the article?

Yes

Is the rationale for developing the new software tool clearly explained?

Yes

Is the description of the software tool technically sound?

Partly

Are sufficient details of the code, methods and analysis (if applicable) provided to allow replication of the software development and its use by others?

Yes

Is sufficient information provided to allow interpretation of the expected output datasets and any results generated using the tool?

Yes

Reviewer Expertise:

Entomology, Microbial Ecology, Microbial systematics, Symbiosis, Bioinformatics, Evolution

I confirm that I have read this submission and believe that I have an appropriate level of expertise to confirm that it is of an acceptable scientific standard.

# Response

**Published:** 2023-02-22

**Authors:** Vincent Hervé

**Affiliations:** INRAE, France

The authors describe SnakeMAGs as a dedicated pipeline for the compositional binning of contigs generated from Illumina sequencing reads obtained from microbial communities. It integrates the various steps in the process of comparative analyses in metagenomes, starting with the assembly of reads generated from community DNA, to the compositional binning of the assembled contigs into high-quality metagenome assembled contigs (MAGs). I believe this pipeline represents a valuable addition to the community. I found the manuscript was easy to read and have the following comments for the authors:

 1. The main advantage of Snake is that it is highly task-specific to the generation of high-quality MAGs from complex communities. It therefore can save considerable time and resources by avoiding steps in processing data (e.g. extensive functional or taxonomic annotations) that are irrelevant to the researcher’s objective.

 I would like the authors to expand on how their pipeline controls for chimeric sequences, since these may lead to an artefactual inflation of MAGs.

**Reply** – Indeed, this aspect was not considered in the first version of our workflow. Therefore, we decided to release a version 1.1 of *SnakeMAGs* that now includes GUNC, a software for detection of chimerism and contamination in prokaryotic genomes. We also evaluated the impact of this tool on the MAGs generated by both ATLAS and *SnakeMAGs.* For the *SnakeMAGs* genomes, we found that 59 out of 65 MAGs, encompassing 13 phyla, passed the GUNC filtering step. With ATLAS, we found that 29 out of 37 MAGs, encompassing 9 phyla, passed the GUNC filtering step. In summary, a few MAGs generated by both workflows did not pass the GUNC quality criteria. Importantly, this new analysis shows that including GUNC does not change the major outcome of our comparison with ATLAS: *SnakeMAGs *produces more MAGs and more diverse MAGs than ATLAS. This analysis has now been included in the revised version of the manuscript.

 2. As a termite researcher, I am aware of the many quirks that termite gut microbiomes present, and the limitations of many data processing pipelines and databases when it comes to addressing those quirks. I was glad that the authors used this challenging community for their benchmarks, and impressed at the contrast between the results from SnakeMAGs and ATLAS. I will emphasize further that the apparent inability of ATLAS to report phyla such as Planctomycetota and Elusimicrobia, or reconstruct fewer MAGs from Fibrobacterota or Desulfobacterota, is not trivial. These bacterial members play major roles in the microbial ecology of the termite gut, and the SnakeMAGs’ ability to detect them highlights its superiority.

 I would like the authors to list/explain some factors could potentially account for this difference.

**Reply** – A potential explanation for this difference can be the relative abundance of the phyla recovered only with *SnakeMAGs *compared to the relative abundance of the phyla recovered by both workflows. We now further discuss this point in the revised version of the manuscript.

 We found no significant difference in the relative abundance (Wilcoxon test, *P* = 0.51) of the MAGs belonging to phyla recovered only with *SnakeMAGs *compared to the phyla recovered by both workflows. However, it should be noted that although *Verrucomicrobiota*, *Planctomycetota*, *Synergistota*, *Elusimicrobiota* and *Acidobacteriota* have been reported among the major taxa in termite gut, they are usually less abundant than *Spirochaetota, Firmicutes* *and* *Bacteroidota *(Arora et al, 2022, *Microbiome*) that have been recovered by both workflows. This result suggests that *SnakeMAGs* is not restricted to the most abundant taxa unlike ATLAS, which could be relatively more prompted to recover only the most abundant microbial taxa.

 3. I found the installation and usage on a Linux Mint server (with comparable processing power to the one used by the authors) to be straightforward. The dependencies are minimalistic and easy to install.

 I congratulate the authors on sharing this tool with the community and hope they find my comments useful.

**Reply** – We thank Reviewer 2 for this positive feedback.

# Peer review: Reviewer response for version 1

**DOI:** 10.5256/f1000research.140650.r158646

**Published:** 2023-01-16

**Authors:** Célio Dias Santos Júnior

**Affiliations:** 1 Institute of Science and Technology for Brain-Inspired Intelligence - ISTBI, Fudan University, Shanghai, China

A method to bin MAGs from reads is described in the publication "SnakeMAGs: a simple, efficient, flexible and scalable approach to rebuild bacterial genomes from metagenomes" by Tradent *et al.* The use of this pipeline to standardize the work with MAGs and make it accessible to batch runs has an obvious benefit. Although the pipeline itself appears a little out of date in terms of conceptual work in the area, I think it has the potential to develop into a useful tool for bioinformatics in general with a few modifications. The critique I offer in this review is more directed at the pipeline's ongoing structural evolution than it is towards the actual article. Although the study reads extremely well, further work must be done to adequately support the use of SnakeMAGs.

 # Abstract

 The authors here should have put greater emphasis on what may be anticipated in terms of the quality of the genomes and species retrieved. It is not favorable if you can recover more genomes but they are of inferior quality, smaller size, or skewed towards a certain species.

 # Methods

 I want to congratulate the authors for the open scientific component. The databases, code, and software were all presented in an organized manner. The documentation reads quite well and appears to be a crucial component of your work. Both the tool's installation and usage are quite well-explained and simple.

 As an adept of collaborative research, I saw that your GitHub lacked some unit tests that would have allowed other contributors to repair bugs that the authors had little interaction with or had previously overlooked without jeopardizing the distribution. That is not a question in this assessment, but it may represent a future enhancement.

 I understand the authors' desire to keep the pipeline short to avoid several dependencies. However, it doesn't appear that the main pipeline is more effective or addresses that issue. For instance, ATLAS is faster, although relying on more dependencies. What benefit do SnakeMAGs have in this regard?

 Despite being condensed, SnakeMAGs does appear to skip certain necessary stages to produce better MAGs, including:

1. Using software that controls for chimera, such as metaMIC ^1^, to fix contigs obtained after assembly;
2. Including multiple binning systems which may seem like a delay but ensures the best bin for each species and even offers a better resolution at the strain level. For instance, using a next-generation binner, such as semiBin or VAMB, then afterward clustering the MAGs on an ANI basis to assure a higher resolution of the species found. Several of these techniques even permit the use of long reads, which may ultimately be advantageous;
3. Since we now know that some of the species binned occasionally display under or overestimated completeness due to bias in the calculations using USCG, run a quality check of the bins using a more comprehensive approach than CheckM, for example, implementing software that addresses contamination, such as GUNC, or even more advanced updates of previous systems that now incorporate machine learning, such as CheckM2 , which does not present bias towards a taxonomy. It would be the best solution for this quality-checking step.

Is there a plan to integrate these actions in the upcoming versions? How may these omitted stages affect the outcomes in the current SnakeMAGs version?

 # Results

 What was the reason for choosing termite gut metagenomes? Is there any indication of better sequencing, deeper sequencing, higher microbial diversity, or any other reason that led the authors to this choice?

 The quality criteria used by authors to classify good genomes (>50% completeness and <10% contamination according to CheckM) is quite loose and not widely adopted. Parks *et al.* (2017) ^2^ - one of the first works using MAGs and also where checkM was presented - used an estimated quality ≥50 (defined as completeness − 5 × contamination). In the present work, if a genome is in the bottom line, it would have an estimated quality of 0, which is far from the threshold Parks adopted. Other criteria are also quite important, e.g. the number of contigs, N50, and ambiguous base pairs. I recommend authors reassess these results using a more strict quality parameter and then report if their results are better or comparable to the other pipelines. I believe that if other binners were combined and similar bins merged as in Parks *et al.* (2017) ^2^, SnakeMAGs would represent a game changer.

 The fact that "SnakeMAGs was able to recover MAGs encompassing a higher diversity of bacterial phyla" is impressive, and I wonder what the average quality of these MAGs recovered in those phyla that ATLAS was not able to bin. Is there any chance that ATLAS discarded bad-quality MAGs that SnakeMAGs is assuming as correct?

 The analysis regarding the memory needed by both systems is missing. The authors mentioned that the peak of memory usage is registered in the SnakeMAGs' logs, and a comparison with ATLAS needs seems important. The longer time for processing, if accompanied by a reduction in memory needs, still seems advantageous in my POV.

 # Discussion

 The fact that the phyla recovered by SnakeMAGs match well with reports of microbial diversity in the samples analyzed are quite appealing to me. However, do the abundances of these species also vary largely? How abundant the phyla recovered only by SnakeMAGs are? Authors should explore this factor to explain if SnakeMAGs' advantage lies in binning genomes from rare species.

 It makes me happy to know that the authors plan versions of the pipeline along with improvements. This spirit of continuous development usually ends up in great bioinformatics tools.

Are the conclusions about the tool and its performance adequately supported by the findings presented in the article?

Partly

Is the rationale for developing the new software tool clearly explained?

Partly

Is the description of the software tool technically sound?

Yes

Are sufficient details of the code, methods and analysis (if applicable) provided to allow replication of the software development and its use by others?

Yes

Is sufficient information provided to allow interpretation of the expected output datasets and any results generated using the tool?

Yes

Reviewer Expertise:

Bioinformatics, microbiology, biochemistry, biotechnology

I confirm that I have read this submission and believe that I have an appropriate level of expertise to confirm that it is of an acceptable scientific standard, however I have significant reservations, as outlined above.

## References

[1] : metaMIC: reference-free misassembly identification and correction of de novo metagenomic assemblies. *Genome Biol* .2022;23(1) : 10.1186/s13059-022-02810-y 242 10.1186/s13059-022-02810-y 36376928PMC9661791

[2] : Recovery of nearly 8,000 metagenome-assembled genomes substantially expands the tree of life. *Nat Microbiol* .2017;2(11) : 10.1038/s41564-017-0012-7 1533-1542 10.1038/s41564-017-0012-7 28894102

# Response

**Published:** 2023-02-22

**Authors:** Vincent Hervé

**Affiliations:** INRAE, France

# Abstract

 The authors here should have put greater emphasis on what may be anticipated in terms of the quality of the genomes and species retrieved. It is not favorable if you can recover more genomes but they are of inferior quality, smaller size, or skewed towards a certain species.

**Reply** – We agree, it is indeed an important point. In the revised version of the manuscript, we show that the quality and genome size of the MAGs recovered by *SnakeMAGs* only did not differ from those belonging to phyla recovered by both *SnakeMAGs* and ATLAS. This is now specified in the abstract of the manuscript.

 # Methods

 I want to congratulate the authors for the open scientific component. The databases, code, and software were all presented in an organized manner. The documentation reads quite well and appears to be a crucial component of your work. Both the tool's installation and usage are quite well-explained and simple.

**Reply** – We thank Reviewer 1 for these positive comments.

 As an adept of collaborative research, I saw that your GitHub lacked some unit tests that would have allowed other contributors to repair bugs that the authors had little interaction with or had previously overlooked without jeopardizing the distribution. That is not a question in this assessment, but it may represent a future enhancement.

**Reply** – We thank Reviewer 1 for this suggestion. We will fully consider it for future enhancement. Meanwhile, users have the opportunity to open Issues on the GitHub page to report any bug or request. Additionally, the test files provided on GitHub allow the user to test our workflow before running real and large datasets.

 I understand the authors' desire to keep the pipeline short to avoid several dependencies. However, it doesn't appear that the main pipeline is more effective or addresses that issue. For instance, ATLAS is faster, although relying on more dependencies. What benefit do SnakeMAGs have in this regard?

**Reply** – Our main goal was to design a simple and minimalist workflow, so a non-specialist can easily bin MAGs without having to choose among (or test and compare) the myriads of software currently available to perform the different steps of our workflow. We believe that the strength of *SnakeMAGs* in this regard is its simplicity. Using *SnakeMAGs*, a non-specialist user just needs to follow general instructions, while using ATLAS, such a user need to make decisive choices of various tools at many steps of the workflow, an approach that can easily become quite confusing.

 Despite being condensed, SnakeMAGs does appear to skip certain necessary stages to produce better MAGs, including:

1. Using software that controls for chimera, such as metaMIC ^1^ , to fix contigs obtained after assembly;
2. Including multiple binning systems which may seem like a delay but ensures the best bin for each species and even offers a better resolution at the strain level. For instance, using a next-generation binner, such as semiBin or VAMB, then afterward clustering the MAGs on an ANI basis to assure a higher resolution of the species found. Several of these techniques even permit the use of long reads, which may ultimately be advantageous;
3. Since we now know that some of the species binned occasionally display under or overestimated completeness due to bias in the calculations using USCG, run a quality check of the bins using a more comprehensive approach than CheckM, for example, implementing software that addresses contamination, such as GUNC, or even more advanced updates of previous systems that now incorporate machine learning, such as CheckM2 , which does not present bias towards a taxonomy. It would be the best solution for this quality-checking step.

Is there a plan to integrate these actions in the upcoming versions? How may these omitted stages affect the outcomes in the current SnakeMAGs version?

**Reply** – We thank Reviewer 1 for these constructive comments. We acknowledge that the processing of metagenomic reads as well as binning are currently very active fields of research and thus, new tools have been published since we started working on our workflow. As mentioned in our Discussion, in the upcoming versions we plan to include alternative software to perform certain tasks and the binning step will be one of them with the addition of SemiBin. CheckM2 is currently in a preprint stage but we also plan to include it as soon as it will be peer-reviewed.

 Regarding the chimera, we felt that it was a significant gap in our workflow so we decided to release a version 1.1 of *SnakeMAGs* that now includes GUNC for genome quality evaluation. We also evaluated the impact of this tool on the MAGs generated by both ATLAS and *SnakeMAGs.* For the *SnakeMAGs* genomes, we found that 59 out of 65 MAGs, encompassing 13 phyla, passed the GUNC filtering step. With ATLAS, we found that 29 out of 37 MAGs, encompassing 9 phyla, passed the GUNC filtering step. In summary, a few MAGs generated by both workflows did not pass the GUNC quality criteria, but including GUNC does not change the major outcome of our comparison with ATLAS: *SnakeMAGs *produces more MAGs and more diverse MAGs than ATLAS. This analysis has now been included in the revised version of the manuscript.

 # Results

 What was the reason for choosing termite gut metagenomes? Is there any indication of better sequencing, deeper sequencing, higher microbial diversity, or any other reason that led the authors to this choice?

**Reply** – It was indeed a deliberate choice to select termite gut metagenomes. We have been working on termite gut microbiota for many years so we are familiar with the microbial diversity present in such systems. Therefore, we have the expertise to properly evaluate the outputs of the workflows from a biological perspective. We would have been less confident with samples from other ecosystems (e.g. deep-sea sediments). Additionally, we would like to mention that termites are well-known to harbor a higher microbial diversity compared to other arthropods. We also selected samples encompassing hosts from different termite families and with different diets, two factors known to impact the gut microbial diversity. Therefore, we believe that termite gut metagenomes constitute relevant datasets to test our workflow.

 The quality criteria used by authors to classify good genomes (>50% completeness and <10% contamination according to CheckM) is quite loose and not widely adopted. Parks *et al.* (2017) ^2^ - one of the first works using MAGs and also where checkM was presented - used an estimated quality ≥50 (defined as completeness − 5 × contamination). In the present work, if a genome is in the bottom line, it would have an estimated quality of 0, which is far from the threshold Parks adopted. Other criteria are also quite important, e.g. the number of contigs, N50, and ambiguous base pairs. I recommend authors reassess these results using a more strict quality parameter and then report if their results are better or comparable to the other pipelines. I believe that if other binners were combined and similar bins merged as in Parks *et al.* (2017) ^2^ , SnakeMAGs would represent a game changer.

**Reply** – Regarding the quality criteria, we used the criteria of “Medium quality MAG” as defined by the Genomic Standards Consortium for the Minimum Information about a Metagenome-Assembled Genome (see Bowers et al, 2017, Nature Biotechnology). This is now specified in the revised version of the manuscript. However, we acknowledge that other authors have used higher quality standard. Following the reviewer’s suggestion, we reassessed our results using the estimated quality ≥50 (defined as completeness − 5 × contamination). As expected, using this higher quality threshold the number of recovered MAGs decreases compared to our initial criteria for both workflows: from 37 to 31 MAGs for ATLAS and from 65 to 46 MAGs for *SnakeMAGs*. Therefore, *SnakeMAGs* still allows the recovery of more MAGs than ATLAS. In terms of diversity, *SnakeMAGs* also recover more phyla ( *n* = 13) than ATLAS ( *n* = 10). In summary, the advantages of our workflow are robust to the MAG quality criteria. These results are now included in the revised version of the manuscript.

 It is noteworthy that in the version 1.1 of *SnakeMAGs* we have now implemented in the Quality assessment step an option to filter the MAGs according to this estimated quality criteria (completeness − 5 × contamination). This will allow users to freely select more stringent quality criteria if they want to.

 The fact that "SnakeMAGs was able to recover MAGs encompassing a higher diversity of bacterial phyla" is impressive, and I wonder what the average quality of these MAGs recovered in those phyla that ATLAS was not able to bin. Is there any chance that ATLAS discarded bad-quality MAGs that SnakeMAGs is assuming as correct?

**Reply** – This is indeed an important point that is now reported in the revised version of the manuscript. Overall, we found no difference in MAG quality or genome size between the two workflows (Wilcoxon test, *P* = 0.15 for completeness; *P* = 0.60 for contamination and *P* = 0.64 for genome size). Regarding the MAGs generated by *SnakeMAGs*, we found no difference in MAG quality or genome size between the phyla also recovered by ATLAS and the phyla only recovered by *SnakeMAGs* (Wilcoxon test, *P* = 0.19 for completeness; *P* = 0.43 for contamination and *P* = 0.19 for genome size). Therefore, we found no evidence supporting the fact that ATLAS discarded bad-quality MAGs that *SnakeMAGs* is assuming as correct. These results are now included in the revised version of the manuscript.

 The analysis regarding the memory needed by both systems is missing. The authors mentioned that the peak of memory usage is registered in the SnakeMAGs' logs, and a comparison with ATLAS needs seems important. The longer time for processing, if accompanied by a reduction in memory needs, still seems advantageous in my POV.

**Reply** – Following Reviewer 1 suggestion, we performed this comparison but found not significant difference in memory usage between the two workflows (Wilcoxon test, *P* = 0.393). This result is now specified in the revised version of the manuscript.

 # Discussion

 The fact that the phyla recovered by SnakeMAGs match well with reports of microbial diversity in the samples analyzed are quite appealing to me. However, do the abundances of these species also vary largely? How abundant the phyla recovered only by SnakeMAGs are? Authors should explore this factor to explain if SnakeMAGs' advantage lies in binning genomes from rare species.

**Reply** – We thank Reviewer 1 for this relevant comment. We now further discuss this point in the revised version of the manuscript. We found no significant difference in the relative abundance (Wilcoxon test, *P* = 0.51) of these MAGs compared to the other phyla. However, it should be noted that although *Verrucomicrobiota*, *Planctomycetota*, *Synergistota*, *Elusimicrobiota* and *Acidobacteriota* have been reported among the major taxa in termite gut, they are usually less abundant than *Spirochaetota, Firmicutes* *and* *Bacteroidota *(Arora et al, 2022, *Microbiome*) that have been recovered by both workflows. This result strongly suggests that *SnakeMAGs* is indeed not restricted to the most abundant taxa.
